# Health Disparities Experienced by People with Disabilities in the United States: A Behavioral Risk Factor Surveillance System Study

**DOI:** 10.5539/gjhs.v4n6p99

**Published:** 2012-09-09

**Authors:** Jennifer R. Pharr, Tim Bungum

**Affiliations:** 1 School of Community Health Sciences, University of Nevada, Las Vegas

**Keywords:** people with disabilities, health care disparities, preventive health services, health services accessibility, Behavioral Risk Factor Surveillance System

## Abstract

The Americans with Disabilities Act became law in 1990; since then research has shown that people with disabilities continue to experience barriers to health care. *The purpose of this study was to compare utilization of preventive services, chronic disease rates, and engagement in health risk behaviors of participants with differing severities of disabilities to those without disabilities.* This study was a secondary analysis of 2010 data collected in the Behavioral Risk Factor Surveillance System national survey in the United States. Rao Chi square test and logistic regression were employed. Participants with disabilities had significantly higher adjusted odds ratios for *all* chronic diseases, for physical inactivity, obesity and smoking. They were significantly more likely to participate in some preventive services (flu/pneumonia vaccination, HIV test) and significantly less likely to participate in other preventive services (mammogram, Pap test). Our findings suggest that people with disabilities are less able to fully participate in all preventive services offered.

## 1. Introduction

### 1.1 Importance of the Problem

The Americans with Disabilities Act (ADA) became a federal civil rights law in 1990 (Americans with Disabilities Act, 2008). The ADA requires that health care providers afford patients with disabilities full and equal access to their health care services and their facility. Additionally, providers must make reasonable modifications as necessary to facilities, policies, and procedures so that health care services are fully accessible to their patients who have disabilities (U.S. Department of Justice & U.S. Department of Health and Human Rights, 2010). Although the ADA prohibits discrimination against people with disabilities in both public and private health care settings, research shows that people with disabilities continue to exhibit health disparities resulting from the absence of health care or the provision of substandard healthcare (Drum, Krahn, Peterson, Horner-Johnson, & Newton, 2009).

Health disparity has been defined by a number of authors and institutions (Braveman, 2006; Carter-Pokras & Baquet, 2002; Health Resources and Services Administration, 2000; Kilbourne, Switzer, Hyman, Crowley-Matoka, & Fine, 2006; Nelson, 2002). By its simplest definition, a health disparity can be described as the preventable, population-specific differences in access to health care, disease presence and health outcomes (Health Resources and Services Administration, 2000). People with disabilities have less access to important preventive health care services (Chan et al., 1999; Cheng et al., 2001; Havercamp, Scandlin, & Roth, 2004; Iezzoni, McCarthy, Davis, & Siebens, 2000; Iezzoni, McCarthy, Davis, Harris-David, & O’Day, 2001; Iezzoni, Davis, Soukup, & O’Day, 2002; Nosek & Howland, 1997; Pharr & Moonie, 2011b; Ramirez, Farmer, Grant, & Papachristou, 2005; Reichard, Stolzle, & Fox, 2011; Thierry, 2000). They are also more likely to report chronic diseases and to engage in more risky health behaviors as compared with people without disabilities (Kinne, Patrick, & Doyle, 2004; Pharr & Moonie, 2011a; Reichard et al., 2011).

Previous research has found that women with disabilities were less likely to receive a Papanicolaou (Pap) test, a breast exam or a mammogram as compared with women without disabilities (Armour, Thierry, & Wolf, 2009; Chan et al., 1999; Cheng et al., 2001; Diab & Johnston, 2004; Havercamp et al., 2004; Iezzoni et al., 2000; Iezzoni et al., 2001; Nosek & Howland, 1997; Pharr & Moonie, 2011b; Ramirez et al., 2005; Reichard et al., 2011; Thierry, 2000). Similarly, women with disabilities were often seen as asexual and were frequently not provided with birth control information (Earle & Church, 2004; Kaplan, 2006; Nosek, Rintala, Young, Foley, & Dunn, 1996). A study by Nosek et al. found that over half of the women with spinal cord injuries had a difficult time finding a physician to manage their pregnancy and that their local hospital could not accommodate them because of mobility limitations (Nosek & Howland, 1997).

In addition to women with disabilities, both men and women with disabilities were less likely to engage in other preventive services and to report good health, and were more likely to report chronic diseases when compared with those without disabilities. People whose disability was severe were less likely to have had their height or cholesterol checked, to have received a tetanus shot, or to have had their teeth cleaned (Armour, Swanson, Waldman, & Perlman, 2008; Havercamp et al., 2004; Iezzoni et al., 2000). Also, they were less likely to engage in physical activity and they were more likely to be overweight compared with those without disabilities (Iezzoni et al., 2000; McGuire, Strine, Okoro, Ahluwalia, & Ford, 2007; Pharr & Moonie, 2011a; Reichard et al., 2011). Adults with disabilities were also more likely to report chronic diseases such as cardiovascular disease (coronary artery disease and stroke), diabetes and asthma (Havercamp et al., 2004; Iezzoni et al., 2002; Pharr & Moonie, 2011a; Reichard et al., 2011).

Although previous studies found that people with disabilities were less likely to have access to some healthcare services and more likely to engage in some health risk behaviors, that relationship is not universal (McGuire et al., 2007; Neri, Bradley, & Groce, 2007; Wei, Findley, & Sambamoorthi, 2006). McGuire et al. found that older US adults with disabilities were more likely to have had a recent flu shot and to have received a lifetime pneumonia vaccination. They also found that adults with disabilities were less likely to smoke. Similarly, Neri and colleagues found that people with disabilities were more likely to have had an HIV test when compared with people without disabilities.

*The majority of previous studies have compared people with disabilities to those without disabilities. Few studies have compared the utilization of preventive services, health risk behaviors or chronic disease rates between people with disabilities of differing severities or degree to those with no disabilities* (Chan et al., 1999; Cheng et al., 2001; Diab & Johnston, 2004; Iezzoni et al., 2000; Iezzoni et al., 2001; Neri et al., 2007). Several studies have utilized data from the Behavioral Risk Factor Surveillance System (BRFSS) survey (Armour et al., 2008; Armour et al., 2009; Diab & Johnston, 2004; Havercamp et al., 2004; Kinne et al., 2004; McGuire et al., 2007; Pharr & Moonie, 2011a; Pharr & Moonie, 2011b). Typically, participants have been identified as having a disability if they answered yes to any of the BRFSS disability questions or if they answered yes to both disability questions (Armour et al., 2008; Armour et al., 2009; Havercamp et al., 2004; McGuire et al., 2007; Pharr & Moonie, 2011a; Pharr & Moonie, 2011b). Diab and Johnson analyzed receipt of preventive health services based on responses between the three BRFSS disability questions in 1998 and 2000; however, in 2001 the BRFSS disability questions were changed, making comparisons with previous data meaningless (Diab & Johnson, 2004).

### 1.2 Aim of the Study

The purpose of this study was to use 2010 national BRFSS data to compare utilization of preventive services, engagement in health risk behaviors and chronic disease rates of participants with three levels of severity of disabilities to participants without disabilities.

## 2. Methods and materials

### 2.1 Behavioral Risk Factor Surveillance System

This study was a secondary data analysis of the 2010 national BRFSS survey. The BRFSS is an annual, random-digit dialing telephone survey that is conducted with non-institutionalized adults (18 years or older) in the United States. The survey is a collaborative effort of the Centers for Disease Control and Prevention and each state (United States, Guam, Puerto Rico and the Virgin Islands). Disproportionate stratified sampling was employed in the survey to provide an adequate sample size for smaller demographic areas (Centers for Disease Control and Prevention, 2010). Data were weighted for population attributes and non-response (Centers for Disease Control and Prevention, 2010). The median response rate for all states in 2010 was 56.4%.

The core component of the BRFSS questionnaire includes questions that are asked of all respondents. These core components include questions about preventive health, chronic diseases and health risk behaviors. Two disability questions were added to the core component of the BRFSS questionnaire in 2001. These questions include: 1) “are you limited in any way in any activities because of physical, mental or emotional problems?” and 2) “do you now have any health problem that requires you to use special equipment such as a cane, a wheelchair, a special bed or a special telephone?” (Centers for Disease Control and Prevention, 2008).

In 2010, 447,997 people completed the disability questions on the national BRFSS survey. Data were analyzed comparing those who answered no to both disability questions to those who answered 1) yes to disability question one only, 2) yes to disability question two only and 3) yes to both disability questions. In 2010, 315,067 participants answered no to both disability questions, 80,833 answered yes to question one only, 12,332 answered yes to question two only and 39,765 answered yes to both disability questions.

### 2.2 Statistical analyses

SAS 9.2 was used for statistical analyses. Weighted descriptive statistics were performed to describe the characteristics of the sample by gender, age, race, education, income, access to health care, check-up in the past year and delaying health care due to cost. To determine statistically significant differences in descriptive statistics between groups, Rao Chi square tests were performed using PROC SURVEYFREQ. Logistic regression was used to calculate crude and adjusted odds ratios for dichotomous dependent variables for: 1) preventive health services, 2) health risk behaviors and 3) chronic disease rates. PROC SURVEYLOGISTIC was used for these analyses. Variables and covariates were selected by perusing previously published literature (American Cancer Society, 2010; Armour et al., 2009; Diab & Johnston, 2004; Iezzoni et al., 2000; Iezzoni et al., 2001; National Cancer Institute, 2010; Pharr & Moonie, 2011a; Pharr & Moonie, 2011b; Reichard et al., 2011; U.S. Preventive Services Task Force, 2010). Covariates included age, income, education, race, gender and access to health care. *When analyzing preventive services utilization, most studies have used a lower age limit for services like mammography (women > 40 years or > 50 years) with no upper age limit* (Armour et al., 2009; Cheng et al., 2001; Diab & Johnston, 2004; Havercamp et al., 2004; Iezzoni et al., 2000; Iezzoni et al., 2001; Pharr & Moonie, 2011b; Reichard et al., 2011). *The U.S. Preventive Services Task Force (USPSTF) recommends upper age limits for some preventive services (Pap test, mammogram, PSA, digital rectal exam and* sigmoid / colonoscopy). For this study, we used the lower and upper age recommendation for preventive services based on the USPSTF. Dependent dichotomous (yes/no) variables included dental cleaning in the past two years, flu vaccine in the past year (any age) and lifetime pneumonia vaccine (men and women 65+), mammogram in the past two years (women 50-74), pap test in the past three years (women 21-65), PSA in the past two years (men 50-75), digital rectal exam in the past two years (men 50-75), sigmoid / colonoscopy ever (men and women 50-75), HIV test (men and women 18-64), physical activity, smoking, binge drinking, obese / overweight, stroke, coronary artery disease, asthma, and diabetes (U.S. Preventive Services Task Force, 2011). The age criteria of preventive services are those of the USPSTF. If age recommendations were not available from the USPSTF, we referred to the CDC, or the National Cancer Institute.

## 3. Results

### 3.1 Characteristics of the Sample

A description of the sample is provided in Table 1. There were significant differences between the four groups for every variable (p < 0.01). Participants with a disability that required the use of special equipment (with and without activity limitations) were slightly more likely to have access to health insurance (90.9% and 92.7%, respectively) and to have had a check-up in the past year (79.4% and 80.6%, respectively), than those without a disability or those with activity limitations only. Those with activity limitations (with and without the use of special equipment) were more likely to delay health care due to cost (22.2% and 24.2%, respectively). Participants with any level of disability were more likely to be in the older age bracket and to be in the lower income bracket compared with participants without disabilities. They were also less likely to be college graduates.

**Table 1:** Descriptive statistics – people with disabilities and people without disabilities

	Without Disabilities (unweighted n=315,067)	Activity Limitations (unweighted n=80,833)	Use Special Equipment (unweighted n =12,332)	Activity Limitations & Special Equip (unweighted n=39,765)
	(weighted % =77.3)	(weighted % = 14.8)	(weighted %=1.9)	(weighted %=6.0)

Viable	Weighted %	Weighted %	Weighted %	Weighted %
Health Insurance*	84.3	84.2	92.7	90.9
Check-up past year*	65.9	68.8	80.6	79.4
Delayed health care due to cost*	12.3	24.2	11.6	22.2

Gender*				
Female	50.5	53.6	49.4	56.1

Age*				
18-24	11.6	6.5	3.6	1.3
25-49	50.9	37.7	22.0	23.3
>50	37.5	55.8	74.4	75.4

Race*				
White	67.6	75.5	67.8	71.3
Black	9.7	8.4	15.3	13.3
Hispanic	15.4	9.9	11.0	8.8
Other	7.3	6.2	5.9	6.6

Income*				
<$20,000	14.5	25.2	28.2	39.8
$20K to <$35K	18.2	22.0	23.7	25.8
$35k to <$75K	30.4	28.2	25.2	22.0
>$75K	36.9	24.6	22.9	12.4

Education*				
< High School Grad	9.3	11.5	17.6	15.5
High School Grad	27.0	29.5	30.9	31.5
Some College	25.4	29.2	23.6	29.5
College Grad	38.3	29.8	27.9	23.5

* = Statistically Significant, Rao-Scott χ^2^ p < 0.01

### 3.2 Preventive Health Services

Crude odds ratios and adjusted odds ratios were calculated for preventive health services, health risk behaviors, and chronic diseases using logistic regression. Variables that were significantly different between groups prior to adjusting for covariates (age, gender, race, income, education and access to health care) remained significant after adjustments, thus only adjusted odds ratios (AOR) are reported in Table 2. When analyses were performed for the comparison of preventive health services by disability status, results were mixed (Table 2). Participants with disabilities age 65 and older were significantly more likely to have had a pneumonia vaccine compared with participants without disabilities age 65 and older (AOR 1.5, 1.8 and 2.3, respectively) and adults with disabilities were significantly more likely to have had a flu vaccine in the past year compared with adults without disabilities (AOR 1.2, 1.8 and 1.7, respectively). Participants with activity limitations (with and without special equipment use) were more likely to have had an HIV test (AOR 1.3 and 1.5, respectively). All participants with disabilities were less likely to have had their teeth cleaned in the past two years (AOR .77, .78 and .58, respectively). Women with activity limitations who also use special equipment were less likely to have had a mammogram in the past two years (AOR .69). Women with activity limitations (with and without special equipment use) were less likely to have had a Pap test in the past three years (AOR .59 and .79, respectively). There was not a significant difference in PSA in the past two years, digital rectal exam in the past two years or sigmoid/colonoscopy ever between the groups with disabilities and those without disabilities

**Table 2:** Health risk behaviors, chronic disease and preventive services - people with disabilities compared to people without disabilities*

	Activity Limitations		Use Special Equipment		Activity Limitations & Special Equip	
	Adj**	Adj**	Adj**	Adj**	Adj**	Adj**
	OR	95% CI	OR	95% CI	OR	95% CI

Dependent Dichotomous Variables						
Preventive Services						
Dental Cleaning	.77	.74 - .81	.78	.72 - .85	.58	.56 - .62
Pneumonia Vaccine	1.5	1.4 - 1.6	1.8	1.7 - 2.0	2.3	2.1 - 2.4
Flu Vaccine	1.2	1.1 - 1.3	1.8	1.6 - 1.9	1.7	1.6 - 1.8
HIV Test Ever	1.3	1.2 - 1.4	1.11	.96 - 1.3	1.5	1.4 - 1.6
Mammogram - 2 yrs	.95	.82 - 1.1	.98	.68 - 1.4	.69	.57 - .84
Pap Test - 3 yrs	.79	.73 - .86	.77	.56 - 1.1	.59	.52 - .67
PSA - 2 yrs	1.5	.98 - 2.2	.90	.51 - 1.6	.66	.34 - 1.3
Digital Rectal Exam – 2 yrs	.99	.92 - 1.1	.96	.92 - 1.1	.91	.82 - 1.0
Sigmoid/colonoscopy	1.3	.95 - 1.7	.96	.58 - 1.6	.96	.67 - 1.4

Health Risks & Chronic Disease						
Smoker	1.6	1.5 - 1.7	.83	.73 - .95	1.5	1.5 - 1.6
Binge Drinker	.86	.80 - .91	.68	.56 - .82	.53	.47 - .61
Physically Inactive	1.7	1.6 - 1.8	1.8	1.7 - 2.0	3.6	3.4 - 3.7

Fair - Poor Health	5.7	5.4 - 6.0	3.6	3.3 - 3.9	14.1	13.3-14.9
Obese	1.8	1.7 - 1.9	2.0	1.8 - 2.2	2.3	2.2 - 2.5
Cancer	1.5	1.3 - 1.6	1.5	1.2 - 1.9	1.9	1.7 - 2.0
Diabetic	1.7	1.6 - 1.8	2.8	2.6 - 3.1	3.2	3.0 - 3.3
CAD	2.6	2.4 - 2.7	2.7	2.4 - 3.0	4.0	3.7 - 4.3
Stroke	2.4	2.3 - 2.7	4.4	3.9 - 5.0	5.6	5.2 - 6.1
Asthma	2.2	2.1 - 2.3	1.9	1.7 - 2.2	3.0	2.8 - 3.2

* = People without disabilities served as the reference group

** = Adjusted for age, income, education, race, gender and access to health care

Logistic regression was used to adjusted odds ratios and adjusted 95% confidence interval

### 3.3 Health Risk Behaviors and Chronic Diseases

Participants with activity limitations (with and without special equipment) were more likely to smoke than participants without disabilities (AOR 1.5 and 1.6, respectively). All participants with disabilities were less likely to binge drink than those without disabilities. All participants with disabilities were more likely to rate their health as fair or poor, to be physically inactive and to be obese compared with participants with no disability. They were also more likely to report a chronic disease (diabetes, coronary artery disease, stroke, asthma and cancer) when compared with participants without disabilities.

## 4. Discussion

### 4.1 Preventive Health Services

Our most important finding was that women with disabilities were less likely to receive important preventive cancer screenings. Women with activity limitations who also use special equipment were less likely to have had a mammogram in the past two years. Women with activity limitations who did not need special equipment and who did need special equipment were less likely to have had a Pap test in the past three years. Although we analyzed the data differently than previous studies, our findings are similar in that groups of women with disabilities are less likely to have had a Pap tests or a mammogram (Armour et al., 2009; Chan et al., 1999; Cheng et al., 2001; Diab & Johnston, 2004; Iezzoni et al., 2000; Nosek & Howland, 1997; Pharr & Moonie, 2011b; Ramirez et al., 2005; Reichard et al., 2011; Thierry, 2000; Wei et al., 2006). This is of concern because participants with disabilities in this study were more likely to report having had cancer and women with disabilities were less likely to have had a Pap test in the past three years. The USPSTF recommends that Pap tests begin for women within three years of the onset of sexual activity or at age 21 (whichever comes first) and that Pap tests continue every three years until the age of 65.

Three reasons may explain the difference in Pap utilization between women with and without disabilities. First, it may have been that the patient and provider decided that the patient no longer required Pap tests because the patient’s risk of cervical cancer was low (i.e. several normal Pap tests in a row and sexual abstinence). Second, the physician may have had a misperception about the patient’s sexual activity and not perceived her to be at risk for cervical cancer (Schopp, Sanford, Hagglund, Gay, & Coatney, 2002; Welner, 1998). This notion is supported by studies that have found that physicians often view women with disabilities as asexual, and as a result fail to offer Pap tests to these patients (Earle & Church, 2004; Kaplan, 2006; M. Nosek et al., 1996; Welner, 1998). Third, women with disabilities may experience physical barriers to Pap tests, particularly, inaccessible exam tables. To be assessed by a Pap test, a patient must be able to transfer themselves or to be transferred by someone else onto an exam table. Studies have found that exam tables that do not lower present a major obstacle for women with disabilities and that a few gynecologists or primary care physicians have height adjustable exam tables (Grabois, Nosek, & Rossi, 1999; Kaplan, 2006; Schopp et al., 2002; Story, Schwier, & Kailes, 2009; Welner, 1998).

In addition to a lower engagement in Pap tests, women with activity limitations who use special equipment were less likely to have had a mammogram in the past two years, and all participants with disabilities were less likely to have had their teeth cleaned. Studies have found inaccessible mammography equipment to be a barrier to mammograms for women with disabilities (Barr, Giannotti, Van Hoof, Mongoven, & Curry, 2008; Mele, Archer, & Pusch, 2005). Additionally, financial challenges and physical accessibility issues (inaccessible dental chairs) have been identified as barriers for people with disabilities to having their teeth cleaned (Rouleau et al., 2011). Because we controlled for income, the lower participation in dental cleaning was most likely due to inaccessible dental chairs. Women in this study who had not had a mammogram also reported that they use special equipment, such as a cane or wheelchair. These findings may help to identify settings in which inaccessible equipment, such as dental chairs or mammography equipment, could produce unequal access to preventive services for those with disabilities; however, this was not specifically addressed in the current study.

Although we found that people with disabilities were less likely to engage in some preventive services based on differing levels of disability, this finding was not consistent for all preventive services. Study participants with disabilities were more likely to engage in some preventive services, such as flu and pneumonia vaccinations, and HIV tests. These findings may be due to participants with disabilities having access to government provided health insurance or being more likely to have had a medical check-up in the past year. A greater number of interactions with their physician could result in an enhanced likelihood that preventive services, such as vaccinations and routine blood work, occur. Additionally, whether a patient has a disability or not, flu / pneumonia vaccinations and HIV tests can be easily administrated. These procedures do not require a patient to transfer to an examination table, or other medical equipment, both of which have been found to be barriers (Becker, Stuifbergen, & Tinkle, 1997; Kroll, Jones, Kehn, & Neri, 2006; Mele et al., 2005; Scheer, Kroll, Neri, & Beatty, 2003; Story et al., 2009). However, each of these preventive measures requires patients to be able to access a health care facility or clinic. These findings suggest that previously cited accessibility issues such as inadequate disability parking (number of spaces or size of spaces), lack of ramps or ramps with steep grades, narrow doorways, heavy doors without automatic opening capabilities, and lack of elevators may be less of an issue for patients with disabilities than are barriers within the physician’s office (Drainoni et al., 2006; Kroll et al., 2006; Scheer et al., 2003; Story et al., 2009). A study by Harrington and colleagues found that only 2.7% of the people with disabilities surveyed reported having a difficult time getting into their primary care physician’s office. However, of the participants who used a wheelchair or were dependent on someone else for transfer, 59% reported being examined in their wheelchair because their primary care physician’s staff were unable to transfer them onto an exam table (Havercamp et al., 2004).

### 4.2 Health Risk Behaviors and Chronic Diseases

After adjusting for covariates, our participants with disabilities had higher odds of reporting chronic diseases (diabetes, asthma, cancer, coronary artery disease and stroke) than participants without disabilities. Participants with disabilities also had greater adjusted odds ratios for being physically inactive, obese and smoking. These finding are consistent with those of previous studies, which show that people with disabilities have significantly more chronic disease, obesity and physical inactivity (Havercamp et al., 2004; Kinne et al., 2004; McGuire et al., 2007; Pharr & Moonie, 2011a; Reichard et al., 2011). While these findings are not surprising, they do raise serious public health concerns. Although we cannot determine causation from this study, the data do show that participants with disabilities have greater risk for adverse health outcomes than those without disabilities (Reichard et al., 2011). Obesity and physical inactivity are two of the major risk factors for chronic diseases such as coronary artery disease, stroke, diabetes, and cancer, and both are modifiable (Nejat, Polotsky, & Pal, 2010; Sothern, Loftin, Suskind, Udall, & Blecker, 1999). Yet, research has found that a majority of health promotion programs have not been modified to meet the unique requirements of people with disabilities (Rimmer & Braddock, 2002; Rimmer, Riley, Wang, & Rauworth, 2005). While the ADA requires that fitness and recreational facilities meet building specification such as an adequate number of accessible parking places, an accessible route into the building and accessible restrooms, it does not specifically require that these facilities have modified equipment, policies, procedures or programs for people with disabilities (Rimmer et al., 2005). Yet, studies have shown that when exercise programs were adapted for people with disabilities there was an increase in physical activity and a decrease in obesity among participants (Kilmer, Wright, & Aitkens, 2005; Olney, et al., 2006, Rimmer, et al., 2009).

Participants with disabilities, especially those who use special equipment, were more likely to be in the older age range and to have health insurance. Advanced age increases the risk of disability. In 2005, 16.5% of people age 21 – 64 reported a disability while 51.8% of those over the age of 65 reported a disability (Brault, 2008). It is estimated that the number of Americans reporting a disability will continue to increase because of the aging of Baby Boomers (people born in the US between 1946 and 1964). In 2011, the first Baby Boomers will reach age 65, yet the median age in America is not anticipated to peak until 2035 (Day, 2005). Americans over the age of 65 and those with a qualifying disability are more likely to have health insurance coverage through Medicare. This may be one explanation for why participants with a disability that required the use of special equipment were more likely to have seen their physician in the past year.

### 4.3 Limitations

There were limitations with this study. Causation cannot be determined because the BRFSS is cross-sectional (Aschengrau, & Seage, 2003). From example, we cannot determine whether the disability caused chronic disease or if chronic disease caused the disability. There was a possibility of bias resulting from self reported information. The participants may also have under or over reported information if they perceived the response to be socially desirable (Adams, Soumerai, Lomas, & Ross-Degnan, 1999). Only household telephones were used in the regular BRFSS sample in 2010. People were excluded from the survey if they were without a home telephone or if they exclusively used a cell phone. There was no direct method for correcting for those who do not have a home telephone, which may have resulted in an underestimation of the true prevalence of disability in this group (Centers for Disease Control and Prevention, 2010). Further institutionalized adults were not included in the BRFSS survey which also may also led to an underestimation of disability.

Specific disability type cannot be determined from the disability questions on the BRFSS survey. For example, people with paraplegia may be at a greater risk for not receiving preventive services than people without paraplegia. However, based on the current BRFSS disability questions, this cannot be determined. Additionally, timing of disability in relation to participation in preventive services could not be established. The preventive service (i.e. sigmoid/colonoscopy ever) may have occurred prior to the person becoming disabled. Despite these limitations, this study contributes to the body of knowledge regarding people with disabilities and health disparities.

## 5. Conclusion

Twenty years after the ADA became law, people with disabilities continue to have unmet health care needs which result in health disparities. Because this study utilized a national data base, it contributes to the limited number of studies assessing the health disparities that people with disabilities experience. People with disabilities were more likely to report all chronic diseases as well as physical inactivity, obesity and smoking. We also found that women with disabilities were less likely to have had important preventive cancer screenings and that participants with disabilities were less likely to have had their teeth cleaned. However, flu and pneumonia vaccinations and HIV tests occurred more often in the groups with disabilities. Our findings suggest that people with disabilities are capable of accessing physicians’ offices but are not able to fully participate in *all* of the preventive services offered. Although the ADA does not specifically require exam tables that lower or other specific accessible medical equipment, the ADA’s 2010 *Access to Medical Care for Individuals with Mobility Disabilities* recommends that physicians’ offices have accessible equipment (U.S. Department of Justice & U.S. Department of Health and Human Rights, 2010). Clearly research is needed to more fully understand the relationship between ADA requirements, and the causes of inaccessibility for health care services among people with disabilities.
